# A Novel T Cell-Engaging Bispecific Antibody for Treating Mesothelin-Positive Solid Tumors

**DOI:** 10.3390/biom10030399

**Published:** 2020-03-04

**Authors:** Aerin Yoon, Shinai Lee, Sua Lee, Sojung Lim, Yong-Yea Park, Eunjung Song, Dong-Sik Kim, Kisu Kim, Yangmi Lim

**Affiliations:** Protein Engineering, Mogam Institute for Biomedical Research, Yongin, Gyeonggi-do 16924, Korea; 33fantasy@hanmail.net (S.L.); lsa0904@mogam.re.kr (S.L.); sjlim95@mogam.re.kr (S.L.); pyy@mogam.re.kr (Y.-Y.P.); potato0224@greencross.com (E.S.); dskim01@mogam.re.kr (D.-S.K.); kisu5347@gmail.com (K.K.); 001lbg03@naver.com (Y.L.)

**Keywords:** mesothelin, CD3, bispecific antibody, solid tumor, cancer immunotherapy, T cell-engaging, heterodimeric bivalent, heterodimeric trivalent, tumor regression

## Abstract

As mesothelin is overexpressed in various types of cancer, it is an attractive target for therapeutic antibodies. T-cell bispecific antibodies bind to target cells and engage T cells via binding to CD3, resulting in target cell killing by T-cell activation. However, the affinity of the CD3-binding arm may influence CD3-mediated plasma clearance or antibody trapping in T-cell-containing tissues. This may then affect the biodistribution of bispecific antibodies. In this study, we used scFab and knob-into-hole technologies to construct novel IgG-based 1 + 1 MG1122-A and 2 + 1 MG1122-B bispecific antibodies against mesothelin and CD3ε. MG1122-B was designed to be bivalent to mesothelin and monovalent to CD3ε, using a 2 + 1 head-to-tail format. Activities of the two antibodies were evaluated in mesothelin-positive tumor cells in vitro and xenograft models in vivo. Although both antibodies exhibited target cell killing efficacy and produced regression of xenograft tumors with CD8+ T-cell infiltration, the antitumor efficacy of MG1122-B was significantly higher. MG1122-B may improve tumor targeting because of its bivalency for tumor antigen. It may also reduce systemic toxicity by limiting the activation of circulating T cells. Thus, MG1122-B may be useful for treating mesothelin-positive solid tumors.

## 1. Introduction

Mesothelin (MSLN) is a 40-kDa glycosylphosphatidylinositol-anchored membrane glycoprotein that is normally expressed primarily on mesothelial cells lining the peritoneum, pericardium, and pleura [1]. It is, however, significantly overexpressed in a number of malignancies, including mesothelioma, ovarian cancer, pancreatic cancer, head and neck cancer, cervical cancer, non-small cell lung cancer, and lung adenocarcinoma, in which it seems to be associated with aggressive phenotypes and a poor prognosis [2,3]. MSLN overexpression in cancers enables tumor-specific targeting using monoclonal antibodies, as well as chimeric antigen receptor (CAR)-T cells containing single-chain variable domain fragments (scFvs) that bind to MSLN [3]. Therefore, MSLN-targeted immunotherapies are being evaluated in phase I and/or phase II clinical trials [4]. However, patients with malignant pleural mesothelioma or ovarian cancer require better systemic treatment, indicating a clear need for the development of novel modalities [5,6].

Bispecific antibodies (bsAbs), which allow for dual targeting, have great potential as therapeutic strategies [7]. Since the concept of bsAbs was originally described by Nisonoff and colleagues more than 50 years ago, technical innovations for generating bsAbs have progressed dramatically. To date, more than 85 bsAbs have been evaluated in clinical trials, and approximately half of all bsAb-related clinical studies have involved T-cell-engaging bsAbs [7]. T-cell bsAbs recruit and engage T cells by binding to both CD3 of the T-cell receptor complex (TCR) and antigen on the target cell, resulting in target cell killing by T-cell proliferation and activation [8,9]. In previous reports, T-cell bsAbs were constructed by combining several anti-CD3 antibodies that showed different affinities and epitopes to the T-cell receptor (TCR). A mucin core protein × CD3εΥ/δε (OKT3) bsAb was constructed for the treatment of bile duct carcinoma, and the antigen-specific cytotoxicity in vitro and inhibition of tumor growth in vivo were investigated [10]. A HER2 × CD3ε (SP34) bsAb specifically killed HER2-expressing cancer cells by T-cell-killing activity and exhibited potent antitumor activity in animal models [11]. In the BCMA × CD3δε (F2B) bsAb format, the anti-CD3δε arm showed low affinity and stimulated low levels of cytokine release, whereas the bsAb demonstrated robust antigen-specific tumor killing both in vitro and in vivo [12]. However, bsAbs that target CD3 have potential safety concerns. Catumaxomab, the pioneering T-cell bsAb, provided important lessons regarding the clinical safety of CD3-targeting antibodies (Abs) [7]. 

Blinatumomab is a CD19 × CD3ε T-cell bsAb that was approved for the treatment of relapsed/refractory B-cell acute lymphocytic leukemia in 2014. It contains two scFvs combined with a flexible linker [13]. Although blinatumomab exhibits highly potent antitumor killing activity, its short serum half-life is a major drawback for clinical applications [8,9], as it must be administered as a continuous intravenous infusion to achieve the desired trough concentrations. IgG-based T-cell bsAbs use a human Ig fragment-crystallized (Fc) region with diminished binding to Fc gamma receptors (FcγRs) to reduce immune effector functions, such as antibody-dependent cellular cytotoxicity or complement-dependent cytotoxicity. However, they maintain binding to neonatal Fc receptors (FcRns) to facilitate IgG recycling [14,15]. 

Generation of bispecific heterodimeric/asymmetric IgG-based antibodies requires the use of several technologies to avoid the random association of heavy and light chains. Correct heavy chain heterodimerization is facilitated using the knob-into-hole (KiH) approach, and correct association of generic light chains is promoted using the common light chain approach or crossmab technology [16,17]. These technologies allow for the construction of various bsAb IgG formats, including asymmetric heterodimeric bivalent 1 + 1 and trivalent 2 + 1 bispecific antibodies, as well as symmetric tetravalent 2 + 2 bispecific antibodies with different valencies [17,18]. Trivalent 2 + 1 IgG antibodies can be generated by fusing a single antigen-binding fragment (Fab) or scFv to the N-terminus of the variable heavy chain (VH) or variable light chain (VL) domain, the C-terminus of the light chain, or the C-terminus of the Fc domain. Similarly, symmetric tetravalent bispecific 2 + 2 antibodies can be generated by fusing Fabs or scFvs via flexible linkers to the N-terminus of the VH or VL domain, the C-terminus of the VL domain, or the C-terminus of the Fc domain of an IgG molecule [17,19]. In the case of T-cell-engaging bsAbs, a number of heterodimeric 1 + 1 or trivalent 2 + 1 IgG bsAbs have been created. For instance, REGN1979 and REGN4018 were generated by using a bivalent 1 + 1 IgG format, with each arm targeting CD20 × CD3 and Mucin 16 × CD3, respectively [20,21]. RG7802, RG6026, EM801, and others were constructed as trivalent 2 + 1 IgG molecules [9,18,22]. As these trivalent antibodies bind only monovalently to the CD3 of TCR chains, TCRs only become cross-linked and activated during concomitant binding of two tumor antigens, resulting in T-cell activation and tumor antigen-dependent T-cell killing of the target cell [9,17,22]. 

Schanzer and colleagues constructed bsAbs using a single-chain Fab-fragment (scFab) to prevent mispairing of light chains with heavy chains [23]. One binding arm was based on scFab, with the light chain attached to the N-terminus of the VH domain by a 32-amino acid (G_4_S)_6_GG linker to form the heavy chain. Dimerization of the two different heavy chains was facilitated by the KiH technology. Upon transient expression, purification yields of these bsAbs were comparable to those of conventional IgG, and the antibodies also exhibited a high level of purity. Thus, the use of two technologies to develop these bsAbs did not affect expression yields or purity. 

In this article, we describe the use of scFab and KiH technology to develop two novel IgG-based bsAbs against MSLN and CD3ε for use as T-cell immunotherapy. These bsAbs had a 1 + 1 or 2 + 1 format. Their antitumor efficacy for MSLN-positive solid tumors was evaluated both in vitro and in vivo.

## 2. Materials and Methods 

### 2.1. Construction of the Antibody Library and Selection of Antibodies Binding to rhMSLN

Mouse immunization was performed in accordance with protocols approved by the Institutional Animal Care and Use Committee of the Green Cross Corporation (#GC-18-014A). Five Balb/C mice were immunized with an initial injection and three booster injections of recombinant human MSLN (rhMSLN; Acro Biosystems, Newark, DE, USA). After the final injection, the animals were euthanized, and total RNA was prepared from spleen tissues. Complementary DNA (cDNA) was synthesized using the ImProm-II reverse transcription system kit (Promega, Madison, WI, USA), according to the manufacturer’s instructions. A phage-displayed mouse/human chimeric Fab Ab library was constructed using the pComb3XTT phagemid vector system, as previously described [24]. Fab clones were selected from the library through four rounds of biopanning, as previously reported [24]. For each round of biopanning, we used 96-well plates coated with 300 ng recombinant human (rh)MSLN protein. After the final round of biopanning, individual phage clones displaying Fab were generated from colonies grown on output plates and tested for reactivity to rhMSLN by using the phage enzyme immunoassay, as previously described [24]. The selected mouse anti-human MSLN antibody was humanized by CDR-grafting methods, based on a previous study [25]. After searching for VH and VL framework sequences in the ImMunoGeneTics database of human germline sequences, IGHV1-3*01 and IGKV1-12*01 were selected as framework templates. Production and purification of anti-MSLN (HMI323) IgG_1_ was performed as previously reported [26].

### 2.2. Generation and Production of anti-CD3ε Antibody 

The mouse anti-human CD3ε antibody SP34 (US2015/016661A1) was humanized by CDR-grafting methods, based on previous reports [27]. We searched for homologous human Abs by using the BLAST sequence program to identify VH and VL framework sequences to use as templates; IGHV3-23*01 and IGLV7-46*01 were selected. The genes for the humanized anti-CD3 antibody were constructed with whole synthesized genes, which were then inserted into the pCI mammalian expression vector (Promega). Production and purification of anti-CD3ε (A15) IgG_1_ were performed as previously described [26]. 

### 2.3. Enzyme-linked Immunosorbent Assay (ELISA)

A total of 250 ng rhMSLN or rhCD3ε (Acro Biosystems) was dissolved in 50 μL phosphate buffered saline (PBS) and added to the wells of a microtiter plate. After incubation overnight at 4 °C and washing three times with PBS containing 0.05% (*v*/*v*) Tween 20 (PBST), the microtiter plate was incubated for 1 h at 37 °C with 1% (*w*/*v*) bovine serum albumin (BSA) in PBS. After washing with PBST, the plate was incubated with 2-fold serially diluted HMI323 IgG_1_ or A15 IgG_1_ protein for 1 h at 37 °C, and then washed three times with PBST. The plate was incubated with anti-human Fab Ab, which was conjugated to horseradish peroxidase (HRP; Sigma, St. Louis, MO, USA) and diluted 5000-fold in PBS. After washing with PBST, 50 μL 3,3,5,5-tetramethylbenzidine substrate solution (Sera Care, Milford, MA, USA) and stop solution (Sera Care) were sequentially added to each well. Optical density was measured at 450 nm using a microtiter plate reader. All tests were conducted in duplicate.

### 2.4. Construction of the Fully Humanized Bispecific Expression Vector

A heterodimeric KiH Fc fragment vector of the human IgG_1_ isotype was constructed as previously described [28]. The construct consisted of two scFabs reactive against MSLN and CD3ε. The heterodimeric 1 + 1 IgG design was monovalent for MSLN and CD3ε, whereas the trivalent 2 + 1 IgG was monovalent for CD3ε and bivalent for MSLN, with one scFab fragment fused to the N-terminus of the CD3ε-specific scFab via (G_4_S)_3_ linkers.

### 2.5. Production and Purification of Bispecific Antibodies

To prepare the 1 + 1 and 2 + 1 bsAb constructs, Expi293 cells were transfected with two expression vectors (knob and hole vectors) using an optimal expression vector ratio. The bsAbs were produced using the Expi293 Expression system (Gibco, Thermo Fisher Scientific, Waltham, MA, USA) [29]. Briefly, plasmid DNA and ExpiFectamin 293 reagent were mixed with Opti-MEM medium and incubated at room temperature for 30 min. The solution was then added to Expi293 cells cultured in Expi293 expression medium and incubated in a shaker incubator at 37 °C with a humidified atmosphere of 8% CO_2_ in air. After incubation for 18 h, ExpiFectamin 293 Transfection Enhancers 1 and 2 were added to each flask, and the transfected cells were incubated under the same conditions for 5 days.

Each bsAb was purified from the cell culture supernatant by affinity chromatography and size exclusion chromatography using PrismA resin and Superdex200 column (GE Healthcare, Chicago, IL, USA), respectively. SDS-PAGE was performed to confirm the size and purity of each bsAb. 

### 2.6. Flow Cytometric Analysis

To perform flow cytometry, we used moderate MSLN-expressing human pancreas adenocarcinoma (AsPC-1), high MSLN-expressing human lung squamous carcinoma (H226), and human T lymphocyte (Jurkat) cell lines. A total of 5 × 10^5^ cells were placed in suspension buffer (2% fetal bovine serum (FBS) containing PBS) and incubated with 5-fold serially diluted heterodimeric 1 + 1 MG1122-A bsAb or trivalent 2 + 1 MG1122-B bsAb for 1 h at 4 °C. After washing with suspension buffer, the cells were incubated for 30 min on ice with phycoerythrin-labeled antibodies against human IgG (Sigma). The cells were subsequently washed, resuspended in 200 μL suspension buffer, and subjected to flow cytometric analysis using the LSRFortesssa cell analyzer (BD Biosciences, San Jose, CA, USA). 

### 2.7. Affinity Evaluation and Dual Binding Assay

Affinity and dual binding activity were evaluated using the Octet system (ForteBio, Fremont, CA, USA) in 96-well microplates at 25 °C, as previously described [30,31]. Briefly, assays were performed by placing aminopropylsilane (APS) Biosensors (ForteBio) nickel-nitrilotriacetic acid (Ni-NTA) or anti-hIgG Fc capture (AHC) in the wells, followed by rinsing in PBS for 60 s, which served as the baseline. The sensors were then immobilized for 300 s with 200 μL rhMSLN or rhCD3ε (20 μg/mL) as antigens and subsequently washed in kinetic buffer (1 mM phosphate, 15 mM NaCl, 0.1 mg/mL BSA, 0.002% Tween-20) for 300 s. The antigen-captured sensors were then submerged in wells containing different concentrations of anti-MSLN (HMI323) IgG_1_ or anti-CD3ε (A15) IgG_1_ monoclonal antibodies as well as MG1122-A or MG1122-B bsAbs for 300 s, followed by 600 s of dissociation in kinetic buffer. Between assays, the sensors were regenerated with 10 mM glycine, pH 1.75. ForteBio Octet analysis software was used to generate the sensorgram and determine the association rate constant (k_a_) and accuracy of the analysis. To analyze the simultaneous binding of the MG1122-A bsAb or MG1122-B bsAb to MSLN and to CD3ε, the antigen-captured sensors were submerged in wells containing 100 nM bsAbs for 300 s, followed by 300–500 s of dissociation in kinetic buffer. Then, the antigen-bsAb-captured sensors were submerged in wells containing 400 nM second antigens for 300 s. The sensors were regenerated as described above. 

### 2.8. T-cell Activation Assay

T-cell activation assays were performed using a Promega kit, following the manufacturer’s protocol. Briefly, 3 × 10^4^ AsPC-1 or H226 cancer cells were seeded into 96-well plates in Roswell Park Memorial Institute (RPMI)-1640 media with 10% FBS. After incubation overnight, the medium was aspirated, and bsAbs and 1 × 10^5^ TCR/CD3 effector cells (NFAT) (Promega) were added. T-cell activation was assessed after 5 h of incubation at 37 °C in 5% CO_2_ atmosphere. Following mixing with Bio-Glo Reagent (Promega), luminescence was measured with a luminescence plate reader (Berthold Technologies, Bad Wildbad, Germany).

### 2.9. Preparation of Peripheral Blood Mononuclear Cells

Leukapheresis products were collected from healthy donors at Samsung Medical Center (SMC) after approval from the SMC institutional review board (No. 2018-01-089). Peripheral blood mononuclear cells (PBMCs) were isolated by centrifugation on a Ficoll density gradient (GE Healthcare) and stored in liquid nitrogen until use.

### 2.10. In Vitro bsAb/hPBMC Cytotoxicity Assay

AsPC-1 or H226 adherent target cells (3 × 10^4^ cells) were transduced with red fluorescent protein using the Incucyte Red NucLight Lentivirus Reagent (Essen BioScience, Ann Arbor, MI, USA). After 24 h, the medium was replaced with fresh growth medium. Puromycin antibiotic was added to obtain a stable, homogenous cell population expressing nucleus-restricted red fluorescent protein. Selected AsPC-1 or H226 cells (1 × 10^4^ cells) were seeded onto 96-well flat-bottom plates. After 24 h, various concentrations of bsAbs (MG1122-A or MG1122-B) or control molecules and human PBMC effector cells were added to the plates at an effector:target ratio of 10:1. All experiments were performed in triplicate. Target cell killing was assessed using the IncuCyte live-cell analysis system after 24 and 48 h of incubation at 37 °C in a 5% CO_2_ atmosphere. The percentage of specific cell lysis was calculated as follows: (effector cells red intensity [RCU × μm^2^/image] when co-cultured with target cells and agent/effector cells red intensity [RCU × μm^2^/image] when co-cultured with only target cells) × 100%. EC_50_ values were calculated using GraphPad Prism5 (GraphPad software, San Diego, CA, USA).

### 2.11. In Vivo Study Using a Tumor Xenograft Mouse Model

All NOG (NOD/Shi-*scid*/IL-2Rγ^null^) mice studies were approved by the Institutional Animal Care and Use Committee of the GC Pharma (No. GC-17-008A). The xenograft mouse study was performed as previously reported [9,32,33]. Female NOG mice (6-8 weeks old; CIEA Japan Inc., Kanagawa, Japan) were subcutaneously injected with AsPC-1 (5 × 10^6^ cells) or H226 (1 × 10^7^ cells) cells. Human T cells were selectively amplified by culturing human PBMCs using Dynabead Human T-Activator CD3/CD28 (Life Technologies, Carlsbad, CA, USA), after which they were injected intraperitoneally into mice as effector cells (effector:target ratio, 2:1) at 5 days after tumor cell implantation. MG1122-A (3 mg/kg, *n* = 5), MG1122-B (3 mg/kg, *n* = 5), or vehicle (PBS, *n* = 5) was administered intraperitoneally 2 days after T-cell transfer and then daily over the next 3–4 days for a total of four injections. Twice per week, the length and width of each tumor were measured with calipers in two perpendicular dimensions, and the tumor volume was calculated using this formula: (width^2^ × length)/2. Clinical signs and body weight were also assessed twice per week. 

### 2.12. Histologic Analysis

Tumor tissue samples were collected 1 week after treatment with bsAbs or PBS. Immunohistochemistry methods were based on previous reports [9,34]. Samples were fixed with formalin and then embedded in paraffin blocks, which were cut into 4-μm sections. Following deparaffinization, the sections underwent heat antigen retrieval and were then stained with human CD3 (anti-CD3, Abcam, Cambridge, UK) or CD8 (anti-CD8, Abcam) using VECTASTAIN Elite ABC kits (VECTOR Lab, Burlingame, CA, USA). The tissues were subsequently counterstained with Mayer’s hematoxylin (Dako, Kyoto, Japan) and examined using an Olympus BX51 microscope (Olympus, Tokyo, Japan). 

### 2.13. Bispecific Antibodies Pharmacokinetics in Nude Mice

All research procedures involving nude mice were approved by the Institutional Animal Care and Use Committee of GC Pharma (No. GC-17-008A). The pharmacokinetics study was performed as previously described [34]. Briefly, 3 mg/kg MG1122-A or MG1122-B was injected through a tail vein of 6- to 8-week-old nude mice (Charles River Japan Lab, Kanagawa, Japan). Blood was then drawn from an intraorbital vein at set times ranging from 5 min to 672 h after injection of the bsAbs. Serum samples were stored at −80 °C. Serum MG1122-A concentrations were measured by sandwich ELISA using CD3ε and biotinylated MSLN (R&D systems, Minneapolis, MN, USA). Serum MG1122-B concentrations were detected using anti-human Fab antibody (Sigma) and HRP-conjugated anti-human Fc antibody (Sigma). 

### 2.14. Statistical Analyses

Continuous variables were compared using two-way analysis of variance, with *p* < 0.01 representing a statistically significant difference between groups. GraphPad Prism (version 5.0) software was used for all statistical analyses. 

## 3. Results

### 3.1. Generation of Anti-MSLN and Anti-CD3 Monoclonal Antibodies

**Figure 1:**
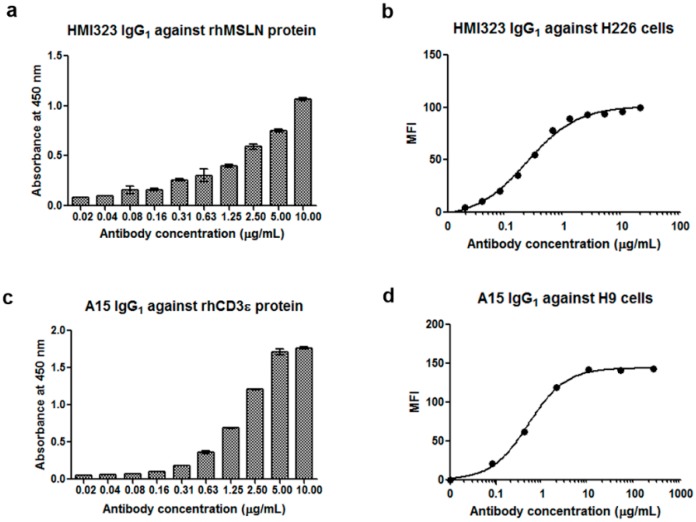
Reactivity of monoclonal antibodies against human mesothelin (MSLN) or human CD3ε. (**a**) Reactivity of HMI323 IgG_1_ against human MSLN protein was investigated by enzyme immunoassay. (**b**) The binding activity of HMI323 IgG_1_ to the MSLN-expressing lung cancer cell line was evaluated by flow cytometry. (**c**) Reactivity of A15 IgG_1_ against CD3ε protein was measured by enzyme immunoassay. (**d**) Binding of A15 IgG_1_ to human T cells was evaluated by flow cytometry.

Mice were immunized with purified rhMSLN. Complementary DNA was synthesized from total RNA that was extracted from the spleen and then used to generate a mouse/human chimeric Fab library containing mouse variable regions and human constant regions with a complexity of 7.5 × 10^8^. After four rounds of biopanning, clones were randomly selected, rescued by infection with helper phage, and subjected to phage enzyme immunoassay to screen for positive clones. MI323, which is a clone reactive to rhMSLN, was selected, and its ability to bind to rhMSLN and the MSLN-overexpressing H226 cell line was confirmed by ELISA and flow cytometry, respectively (data not shown). The MI323 clone was then humanized by grafting the CDRs of VH and VL to IGHV1-3*01 and IGKV1-12*01 templates, respectively. After expression of the humanized clone HMI323 IgG_1_ in mammalian cells and subsequent purification, binding activity was confirmed by ELISA and flow cytometry (Figure 1a,b). HMI323 IgG_1_ bound to the rhMSLN protein and H226 cell line in a dose-dependent manner. 

To generate the CD3 monoclonal antibody, we used the mouse anti-human CD3ε antibody SP34 as a template. The SP34 clone was humanized using the IGHV3-23*01 and IGLV7-46*01 framework templates. Finally, we obtained a clone (A15) that reacted to the CD3ε protein. A15 IgG_1_ was then expressed in a mammalian cell line and purified using a protein A column. ELISA confirmed that A15 IgG_1_ bound to CD3ε protein in a dose-dependent manner (Figure 1c). A15 IgG_1_ binding to the CD3-expressing cell line was confirmed by flow cytometry, and strong reactivity of A15 IgG_1_ was observed with CD3-positive H9 cells (Figure 1d). 

### 3.2. Construction of Anti-MSLN/CD3 Bispecific Antibodies

**Figure 2:**
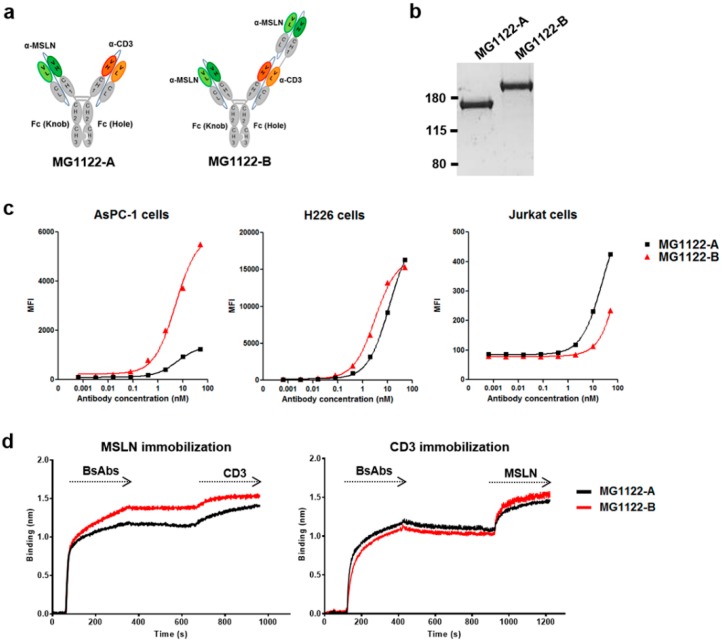
Structural features and binding of bispecific antibodies. (**a**) Structural characteristics of bispecific antibodies MG1122-A and MG1122-B. MG1122-A is an asymmetric IgG_1_-based human antibody against MSLN and CD3ε. MG1122-B is an asymmetric two-arm IgG_1_ human antibody, bivalent against MSLN and monovalent against CD3ε. (**b**) Purity and molecular weight of the antibodies were assessed by SDS-PAGE analysis. (**c**) Specific binding of bispecific antibodies to MSLN in two MSLN-expressing cancer cell lines (AsPC-1^moderate^ and H226^high^) and to CD3 in the Jurkat cell line was evaluated by flow cytometry. (**d**) Dual antigen binding of bispecific antibodies to MSLN and CD3 was measured by the Octet system.

MG1122-A (1 + 1 format) (i.e., HMI323/A15) was generated by combining two scFabs: one from the anti-MSLN antibody HMI323, and one from the anti-CD3ε antibody A15. A novel trivalent MG1122-B (2 + 1 format) was constructed by fusing another HMI323 scFab to the N-terminus of the VL domain of A15 scFab (Figure 2a). Heavy chain association was facilitated during the generation of asymmetric bsAbs via the KiH technology. To minimize binding to FcγRs and complement component C1q, we used L234A and L235A mutations on the heterodimeric Fc. Bispecific Abs were produced by co-transfecting Expi293F cells with the mammalian expression vectors using ExpiFectamin 293 reagent. The protein secreted into the supernatants was purified by protein A affinity chromatography, followed by size exclusion chromatography. Purity of the bsAbs was confirmed by SDS-PAGE and Coomassie blue staining (Figure 2b). The calculated molecular weight was approximately 149.9 kDa for MG1122-A and 199.9 kDa for MG1122-B. The purity was high for both MG1122-A and MG1122-B; however, the average yield of MG1122-A was higher than that of MG1122-B (29.6 ± 1.4 mg vs. 5.14 ± 1.5 mg), due to the fact that the molecular mass on the hole arm of MG1122-B was much higher than that of MG1122-A.

To investigate the MSLN protein expression profile in cancer cell lines, we performed flow cytometry using an anti-hMSLN rat antibody (R&D systems). MSLN-positive cell lines exhibited varying levels of MSLN expression (Figure S1). We selected the moderate MSLN-expressing pancreas adenocarcinoma line (AsPC-1) and the high MSLN-expressing lung squamous cell carcinoma line (H226) for further study. We used flow cytometry to confirm the dual binding activity of bsAbs to MSLN-positive cell lines and the CD3ε-expressing Jurkat T-cell line. Both anti-MSLN/CD3 bsAbs reacted to AsPC-1, H226, and Jurkat T cells (Figure 2c). When AsPC-1 or Jurkat cells were exposed to high concentrations of Ab, differences in MFI were observed between MG1122-A and MG1122-B. The reactivity of MG1122-B to the moderate MSLN-expressing cell line was higher than that for MG1122-A, whereas the reactivity of the two anti-MSLN/CD3 bsAbs to the high MSLN-expressing cell lines was similar. The binding activity to CD3ε was lower for MG1122-B than for MG1122-A, presumably because the CD3ε binding region of MG1122-B was introduced at an “inside” position. Similar findings were observed with the apparent equilibrium dissociation constant (K_D_) values for the binding of bsAbs to rhMSLN and CD3ε protein. The binding affinities of the parental monoclonal IgGs (HMI323 and A15) were 4.87 and 1.17 nM, respectively (Table 1). For bsAbs, MG1122-A bound to rhMSLN or CD3 with a K_D_ of 94.3 nM or 14.1 nM, respectively. MG1122-B bound to rhMSLN or CD3ε with a K_D_ of 7.79 nM or 52.6 nM, respectively. Because MG1122-B was bivalent for MSLN, MG1122-B bound to MSLN with an affinity that was similar to that of the parental HMI323 IgG. The binding affinity to CD3 was lower for MG1122-B than for MG1122-A, which was a similar result of the binding activity to Jurkat T cells. 

To evaluate whether anti-MSLN/CD3 bsAbs are capable of binding simultaneously to MSLN and CD3, we measured “sandwich” experiments by using the Octet system (Figure 2d). Both anti-MSLN/CD3 bsAbs bound simultaneously to MSLN and CD3. Interestingly, differences in binding activity were also observed between MG1122-A and MG1122-B. The reactivity of MG1122-B to MSLN was higher than that of MG1122-A, whereas the reactivity of MG1122-B to CD3 was lower than that of MG1122-A.

### 3.3. Characterization of In Vitro Activity of anti-MSLN/CD3 Bispecific Antibodies

**Figure 3:**
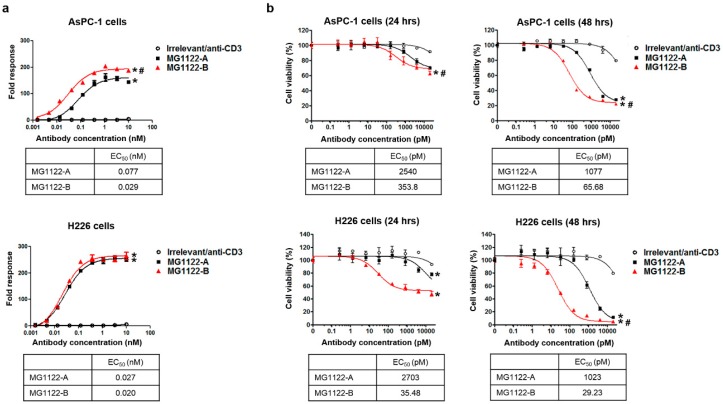
T-cell activation and cytotoxic activity of bispecific antibodies against cancer cell lines. (**a**) Bispecific antibody-mediated, T-cell activation was measured by incubating Jurkat-NFAT-luc cells with AsPC-1 or H226 cancer cells in the absence or presence of increasing concentrations of bispecific antibodies. RLU, relative luminescence units corresponding to the intensity of luciferase expression downstream of CD3. (**b**) Dose-dependent tumor cell lysis was detected at 24 and 48 h after incubation of bispecific antibodies with human peripheral blood mononuclear cells and tumor cells (effector:target ratio, 10:1). * *p* < 0.01 vs. irrelevant/anti-CD3, # *p* < 0.01 vs. MG1122-A.

To investigate the MSLN-specific T-cell activity of anti-MSLN/CD3 bsAbs, we used the genetically engineered cell line (TCR/CD3 effector cells; NFAT). When these cells were exposed to an anti-TCR/CD3 stimulus, receptor-mediated signaling induced luminescence via activation of NFAT. We incubated TCR/CD3 effector cells with MSLN-positive target cells in the absence or presence of increasing concentrations of anti-MSLN/CD3 bsAbs or irrelevant/anti-CD3 (anti-glypican3/CD3 bsAb; manuscript in preparation). Our results show that anti-MSLN/CD3 bsAbs significantly induced MSLN-specific T-cell activation in a dose-dependent manner, whereas a control bsAb (irrelevant/anti-CD3) had no effect (Figure 3a). More importantly, MG1122-B increased the T-cell activation of AsPC-1 target cells with a 2- to 3-fold stronger half-maximum effect (lower EC_50_) than MG1122-A. MG1122-B induced significantly more T-cell activation than MG1122-A. These results indicate that binding between T cells and MSLN-positive cancer cells by anti-MSLN/CD3 bsAbs leads to TCR/CD3 crosslinking and activation of the CD3 downstream signaling pathway. 

Next, we used cytotoxicity assays to determine the ability of anti-MSLN/CD3 bsAbs to promote the antigen-dependent lysis of AsPC-1 and H226 targets. As shown in Figure 3b, induction of the CD3 downstream signaling pathway anti-MSLN/CD3 bsAbs allowed us to measure T-cell-induced killing of MSLN-positive cancer cell lines at 24 and 48 h of co-culturing PBMCs and MSLN-positive cancer cells at an effector:target ratio of 10:1. Both anti-MSLN/CD3 bsAbs significantly induced the T-cell-mediated lysis of MSLN-positive AsPC-1 and H226 cells in a concentration-dependent manner, whereas a control bsAb had no effect. T-cell-mediated lysis of target cells was evident at 24 h and was almost 80% or higher at 48 h. These results suggest that cross-linkage of T cells with MSLN-positive tumor cells via the CD3 complex using anti-MSLN/CD3 bsAbs results in efficient activation of T cells and tumor cell lysis. Although both bsAbs directed T cells to kill the tumor cells with high potency, the killing effect at 48 h was greater with MG1122-B than with MG1122-A, with an approximately 16- to 35-fold higher apparent potency. Our results indicate that bivalent binding to target tumor cells leads to enhanced T-cell activation and T-cell-mediated target cell lysis.

### 3.4. In Vivo Antitumor Effects of Anti-MSLN/CD3 Bispecific Antibodies in a Tumor Xenograft Mouse Model

**Figure 4:**
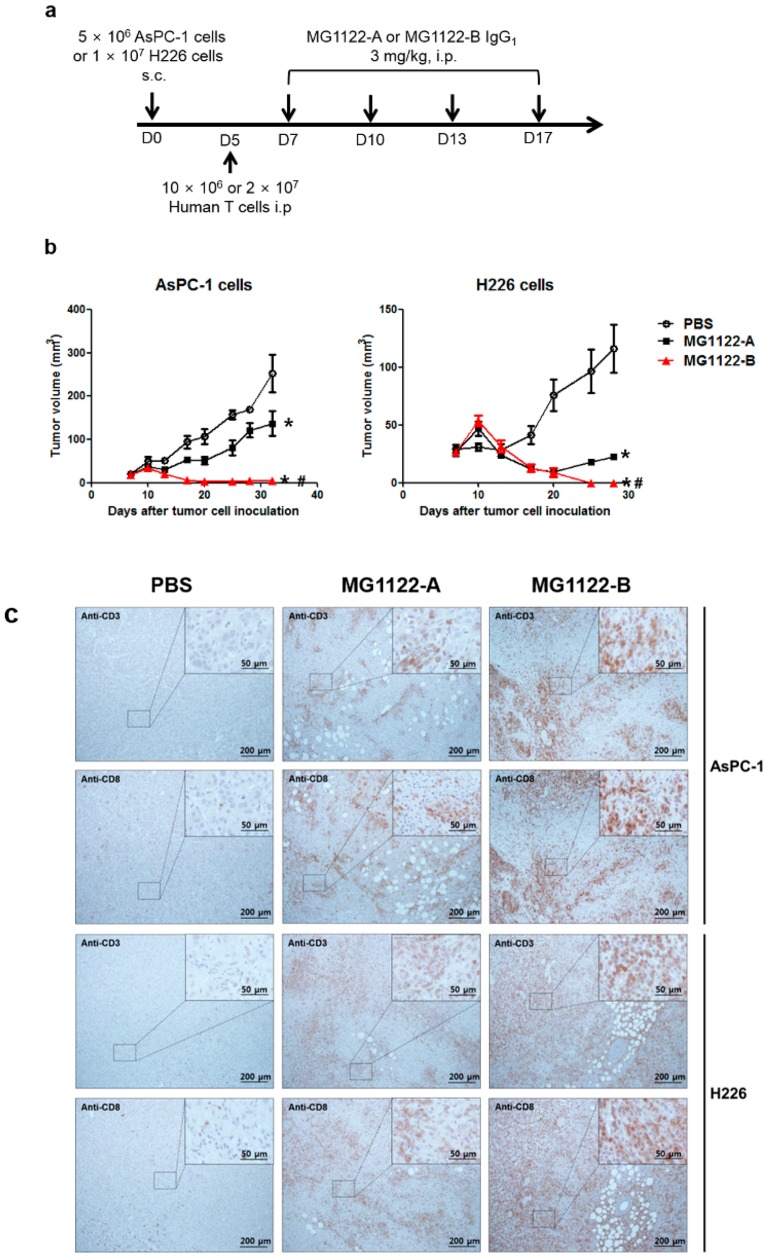
In vivo efficacy of bispecific antibodies in the xenograft model. (**a**) Schematic depiction of the antibody treatment protocol. Human T cells were injected intraperitoneally (effector:target ratio, 2:1) into NOG mice at 5 days after the injection of AsPC-1 or H226 tumor cells. Mice bearing xenograft tumors were treated with MG1122-A or MG1122-B at a dose of 3 mg/kg twice per week, starting at day 7 after tumor cell injection. (**b**) Tumor growth was measured twice per week for the indicated time periods. Data points represent the mean ± standard deviation (SD) of five animals. * *p* < 0.01 vs. PBS, # *p* < 0.01 vs. MG1122-A. (**c**) Representative histologic images of tumor sections stained for human CD3 and CD8 (all brown). Scale bars are shown on each panel. Representative images of at least three mice were analyzed in each group.

In vivo antitumor activity was evaluated in NOG mice with AsPC-1 or H226 subcutaneous xenografts. Because A15 IgG has no cross-reactivity with mouse CD3, human T cells were injected intraperitoneally into the mice on day 5, after which MG1122-A, MG1122-B, or PBS (vehicle) was administered twice per week for 2 weeks at a dose of 3 mg/kg (Figure 4a). Body weights of the mice (measured twice per week) did not differ between the anti-MSLN/CD3 bsAb–treated groups and control group (data not shown). Tumor growth inhibition was observed after the second intraperitoneal injection in the anti-MSLN/CD3 bsAbs treatment groups (Figure 4b). Control PBS-treated mice had significantly higher tumor volumes compared to anti-MSLN/CD3 bsAbs–treated mice. Notably, MG1122-B induced complete tumor regression in both AsPC-1 and H226 xenograft models. MG1122-A was significantly less effective at inhibiting tumor growth than MG1122-B and was more effective in the H226 model than in the AsPC-1 model. The latter results are consistent with the specificity of MG1122-A, reflecting its greater potency against high MSLN-expressing H226 cells. Furthermore, its poor activity in the AsPC-1 xenograft model suggests a lack of efficacy of monovalent anti-MSLN therapeutics in this model and the necessity for the dual MSLN-targeting arm. 

The in vivo efficacy of anti-MSLN/CD3 bsAbs was further confirmed by immunohistochemically examining the levels of tumor-infiltrating CD3 and CD8 T cells in formalin-fixed, paraffin-embedded syngeneic tumor tissues at day 13 (Figure 4c). Histologic staining revealed prominent infiltration of T cells within the tumors in both anti-MSLN/CD3 bsAbs treatment groups, whereas tumors from vehicle-treated mice had less T-cell infiltration. Relocalization of CD3 and CD8 T cells upon MG1122-B treatment was greater in both xenograft models, compared with vehicle treatment. The abundant CD8+ lymphocytes in tumor tissues provides a theoretical foundation for the clinical adoption of an immunotherapy strategy targeting MSLN and CD3.

### 3.5. Anti-MSLN/CD3 Bispecific Antibodies Pharmacokinetics In Vivo

**Figure 5:**
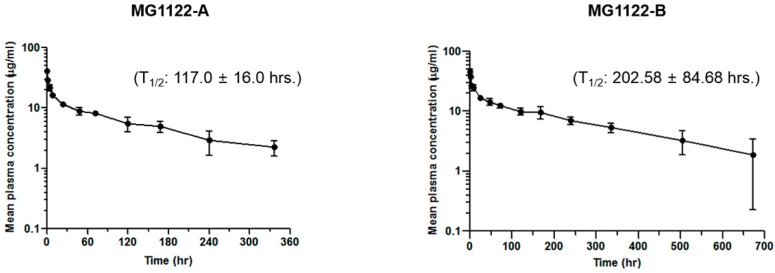
Serum half-life of bispecific antibodies. Serum concentrations of bispecific antibodies after injection of 3 mg/kg into six nude mice. Blood was sequentially collected at 5 min to 336 or 672 h after injection to measure serum MG1122-A or MG1122-B IgG concentrations, respectively. Each point represents the mean ± SD of six mice. i.v., intravenous injection.

In vivo pharmacokinetic single-dose studies were performed in nude mice. After the mice were injected intravenously with 3 mg/kg anti-MSLN/CD3 bsAbs, blood samples were collected at 5 min and at 1, 4, 8, 24, 48, 72, 120, 168, 240, and 336 h post-injection. Blood samples were also obtained from MG1122-B–treated animals at 504 and 672 h. Plasma concentrations of bsAbs were determined using the sandwich ELISA assay. The semilogarithmic plots of mean plasma concentration–time curves for both anti-MSLN/CD3 bsAbs are shown in Figure 5, and the plasma concentrations and main pharmacokinetic parameters are reported in Supplementary Table S1. The half-life of MG1122-A was approximately 117 h, and the half-life of MG1122-B was approximately 202.58 h. The maximum serum concentration (C_max_) of MG1122-B was 45.46 μg/mL, and the C_max_ of MG1122-A was 41.4 μg/mL. The area under the curve from time 0 to infinity (AUC_inf_; a measure of systemic exposure) and total clearance (CL) of MG1122-B were 5270.89 μg.h/mL and 0.61 mL/h/kg, respectively, whereas the AUC_inf_ and CL of MG1122-A were 2296 μg.h/mL and 1.31 mL/h/kg, respectively. Collectively, these results suggest that the 2 + 1 format decreases clearance and increases systemic exposure in mice.

## 4. Discussion

The development of bsAbs has received considerable attention in the past 20 years [35,36]. For treatment in oncology, one bsAb has received marketing approval, and more than 50 other bsAbs are undergoing clinical trials. These bsAbs have varying formats and mechanisms of action. Several previous studies presented evidence that the bsAb structure contributes significantly to the activity of these molecules. Most bsAbs recruit immune cells to kill tumor cells. 

In the research described in this report, we generated two novel T-cell-engaging bsAbs for targeting MSLN-expressing solid tumors, using scFab and KiH technologies to prevent random association of heavy and light chains. MG1122-A is a heterodimeric 1 + 1 bispecific IgG molecule, with each antigen-binding fragment (scFab) targeting MSLN and CD3ε. MG1122-B is an asymmetric 2 + 1 bispecific antibody, which was constructed by head-to-tail fusion of the MSLN and CD3ε-binding Fab domains via a flexible linker. MG1122-B exhibits bivalent binding to MSLN and monovalent binding to CD3ε. The use of a bivalent tumor-targeting arm may enhance potency and tumor selectivity through enhanced binding avidity [37]. Three 2 + 1 bsAbs—anti-CEA/CD3 (RG7802), anti-CD20/CD3 (RG6026), and anti-BCMA/CD3 (EM801)—were previously generated using crossmab and KiH technologies [9,17,22]. They bind monovalently to CD3 with low affinity and bivalently to target cells with higher avidity to facilitate preferential target cell binding. Similarly, the bivalency of MG1122-B for tumor antigen promotes high binding avidity toward tumor cells, and its tumor targeting was superior to that of MG1122-A, which binds monovalently to MSLN. MG1122-B also exhibited better binding to tumor cells with high or moderate expression. The high-avidity binding to MSLN led to an enhanced selective killing of both moderate MSLN-expressing and high MSLN-expressing tumor cells both in vitro and in vivo. These findings suggest that MG1122-B could be useful for treating a wide variety of MSLN-expressing cancers. In contrast, MG1122-A activity was more dependent on MSLN expression, with higher in vivo potency observed in high MSLN-expressing tumors than in moderate MSLN-expressing tumors. 

The administration of anti-CD3 antibody may produce acute systemic toxicity in both humans and mice; this has been attributed to cytokine release following antibody-induced T-cell activation [38]. Several studies have demonstrated that the affinity of anti-CD3 antibodies dramatically affects the biodistribution of bsAbs [7]. Achieving optimal T-cell cytotoxicity requires optimal kinetics for CD3 binding [39], and high-affinity anti-CD3 molecules do not necessarily lead to enhanced T-cell activation. Lower-affinity anti-CD3 antibodies are preferable to facilitate good tumor distribution in vivo, without CD3-mediated plasma clearance or binding of the antibody within T-cell-containing tissues, such as the spleen and lymph nodes [40,41]. In previous reports, anti-CD3 antibodies have exhibited affinities ranging from 1 to 200 nM, as demonstrated by surface plasmon resonance. For instance, the anti-CD3 affinities of blinatumomab and AFM11 were 100 and 70 nM, respectively [42]. Head-to-tail geometry in a 2 + 1 format, in which the CD3ε binding region is introduced in an “inside” position, may increase the biodistribution of bsAbs by reducing CD3 affinity. For example, the anti-CD3 affinities of the head-to-tail 2 + 1 bsAbs RG7802 and EM801 were 100 nM and 70–100 nM, respectively [9,22]. Both RG7802 and EM801 produce minimal nonspecific T-cell activation in the absence of target cells. In clinical trials, anti-CEA/CD3 (RG7802) and EM801 exhibited manageable toxicities [22,43]. MG1122-A was generated by combining anti-MSLN scFab on the knob arm and anti-CD3 scFab on the hole arm. MG1122-B was constructed by fusing another anti-MSLN scFab to the N-terminus of anti-CD3 scFab via (G_4_S)_3_ linkers. The CD3ε-binding region of MG1122-B was also introduced in an “inside” position, reducing the affinity to the CD3-binding arm compared with the affinity of MG1122-A. Therefore, MG1122-B may be less likely to bind to nonspecific T cells, leading to less systemic toxicity by avoiding the undesired activation of T cells in peripheral blood and less trapping of the antibody in the spleen or lymph nodes. However, when MG1122B reaches the target cells, its efficacy might be attributed to flexible tumor-targeting scFab arms via a flexible linker. This linker may allow for increased binding chances between the antibody and tumor and enhanced affinity to a potent CD3-targeting arm that can bind and activate T cells more easily at the tumor site, because of conformational changes in the tumor-targeting scFab upon binding.

MSLN is an attractive target for cancer therapy, and a growing number of clinical trials are currently exploring diverse MSLN-targeted strategies, including the use of immunotoxin, antibody drug conjugates, monoclonal antibodies, and CAR-T cells. The anti-MSLN monoclonal antibody amatuximab (also called MORAB-009) binds to MSLN and induces antibody-dependent, cell-mediated cytotoxicity. Phase II clinical trials have been conducted with amatuximab treatment alone or in combination with pemetrexed and cisplatin [44]. Phase I studies with immunotoxin SS1P or the antibody drug-conjugate BAY 94-9343 have also been performed. Immunotoxin SS1P is limited by the development of neutralizing antibodies specific for the toxin portion and possibly also for the chimeric SS1 antibody [4]. MG1122-A and MG1122-B are expected to have less immunogenicity, because humanized antibodies are associated with a lower risk of immune responses in humans than chimeric antibodies [45]. Several phase I clinical trials have been initiated to determine the safety and maximally tolerated dose of MSLN CAR-T-cell therapy, but on-target/off-tumor toxicity is a concern. Moreover, the solid-tumor microenvironment provides several obstacles for MSLN CAR-T-cell therapy, and MSLN CAR-T cells must overcome immune barriers to infiltrate tumors. Our in vivo results demonstrating antitumor efficacy through T-cell infiltration of the solid-tumor microenvironment suggest that MG1122-A and MG112-B may be superior to CAR-T therapy for solid tumors. Other strategies have involved the production of bsAbs targeting MSLN and engaging other types of immune cells. For example, Bano and colleagues generated a Fab-like bsAb targeting MSLN and FCγRIII (CD16) that is expressed by natural killer cells [1]. Similarly, Ye and colleagues constructed bsAbs containing single-chain Fv domains against MSLN and CD40 [46]; to avoid systemic toxicity, their bsAbs had agonistic anti-CD40 activity. Notably, by limiting the antigen sink effect of CD3 in the periphery, MG1122-B was designed as an MSLN-targeting, T-cell-engaging bsAb with low systemic toxicity.

Recently, resistance to bsAb treatment has been observed in clinical trials [7]. Downregulation of bsAb-specific, tumor-associated antigens on tumor cells is one potential mechanism of tumor escape, whereas other mechanisms may involve immune suppression by regulatory T cells or immune checkpoint molecules. For example, programmed cell death 1 (PD-1) and programmed cell death 1 ligand 1 expression, which may be induced by bsAbs, may limit the activity of T-cell bsAbs. Accordingly, combining a T-cell bsAb with a PD-1 antibody has been shown to enhance antitumor activity [7]. Hence, combining MG1122-B with an immune checkpoint inhibitor may be a useful strategy for improving antitumor efficacy. 

## 5. Conclusions

MG1122-A and MG1122-B are novel IgG-based, T-cell-engaging antibodies for targeting MSLN-expressing solid tumors. Both Abs were constructed using scFabs and KiH technologies. MG1122-A is an asymmetric bivalent 1 + 1 bsAb that is reactive against MSLN and CD3, whereas MG1122-B is an asymmetric trivalent 2 + 1 bsAb that is monovalent for CD3 and bivalent for MSLN. Both bsAbs mediate crosslinking of MSLN on target cells with CD3 on T cells, leading to effector T-cell-dependent lysis of the target in vitro and in vivo. However, the two Abs differ, with MG1122-B exhibiting higher avidity for tumor antigen and greater efficacy for T-cell activation and tumor killing. Furthermore, the CD3ε binding region of MG1122-B was introduced in an “inside” position, which may reduce the affinity of the CD3-binding arm and thereby decrease systemic toxicity by limiting undesired activation of T cells in the periphery. Further studies are required to more fully understand MG1122-B, including its effects on cytokine secretion and its mechanism of action. Overall, our results suggest that using the MG1122-B bsAb to promote T-cell activity against MSLN-expressing tumor cells could be a useful treatment strategy for solid tumors.

## 6. Patents

Mogam Institute for Biomedical Research and GC Pharma have filed patent applications related to this work for anti-CD3 monoclonal, anti-MSLN monoclonal, and anti-MSLN/CD3 bispecific antibodies.

## Figures and Tables

**Table 1:** Affinity of monoclonal and bispecific antibodies to human MSLN and CD3.

Antigen	Clone	k_a_ (1/Ms)	k_d_ (1/s)	K_D_ (nM)
MSLN	HMI323	3.24 × 10^5^	1.58 × 10^−3^	4.87
	MG1122-A	2.04 × 10^4^	1.92 × 10^−3^	94.3
	MG1122-B	9.94 × 10^4^	7.74 × 10^−4^	7.79
CD3	A15	3.90 × 10^5^	4.57 × 10^−4^	1.17
	MG1122-A	6.93 × 10^5^	9.76 × 10^−3^	14.1
	MG1122-B	4.61 × 10^5^	2.42 × 10^−2^	52.6

## Supplementary Materials

Supplementary Figure S1 and Table S1 are available online at https://www.mdpi.com/2218-273X/10/3/399/s1, Figure S1: title, Table S1: title.
