# Supplementary material for: A Novel T Cell-Engaging Bispecific Antibody for Treating Mesothelin-Positive Solid Tumors
Source: Biomolecules. 2020 Mar 4;10(3):399. doi: 10.3390/biom10030399 (PMC7175222; doi:10.3390/biom10030399)
Supplement: Supplementary file 1 [file biomolecules-10-00399-s001.pdf]

biomolecules

MDPI

Article

A Novel T Cell-Engaging Bispecific Antibody for Treating Mesothelin-Positive Solid Tumors

Aerin Yoon *, Shinai Lee, Sua Lee, Sojung Lim, Yong-Yea Park, Eunjung Song, Dong-Sik Kim, Kisu Kim and Yangmi Lim

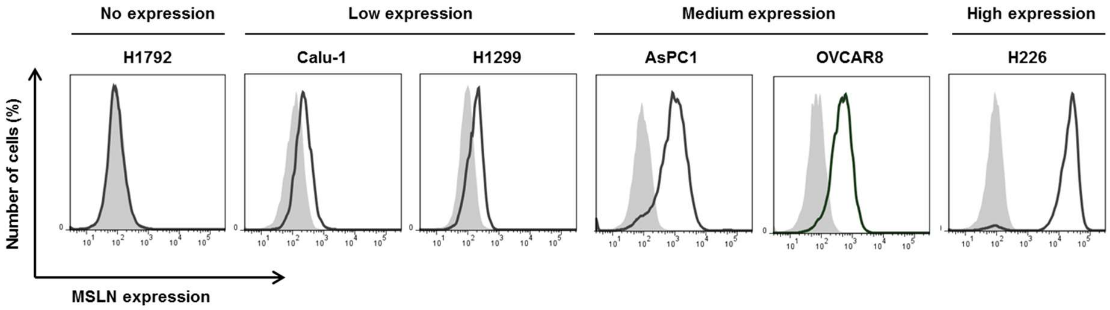

Supplementary Figure S1. MSLN protein expression in representative cell lines. The level of MSLN expression was determined by flow cytometry. Gray without anti-MSLN antibody; black with anti-MSLN antibody**.**

Supplementary Table S1. Pharmacokinetic parameters of anti-MSLN/CD3 bsAbs

MG1122-A			Parameter (units)	MG1122-B		
3			Dose (mg/kg)	3		
117	±	16.0	$T_{1/2}$ (h)	202.58	±	84.68
41.4	±	3.11	C_{max} (μg/mL)	45.46	±	5.52
1939	±	123	$AUC_{0\text{-}t}$ (μg.h/mL)	4568.32	±	821.75
2296	±	149	$AUC_{0\text{-}inf}$ (μg.h/mL)	5270.89	±	1719.18
15.5	±	3.06	AUC_%Extrap_obs (%)	10.65	±	11.09
1.31	±	0.081	CL (mL/hr/kg)	0.61	±	0.17
170	±	12.9	MRT (hr)	210.51	±	30.99
222	±	10.1	Vss (mL/kg)	166.09	±	19.28
